# Relationship between the Young's Modulus and the Crystallinity of Cross‐Linked Poly(ε‐caprolactone) as an Immobilization Membrane for Cancer Radiotherapy

**DOI:** 10.1002/gch2.202000008

**Published:** 2020-06-17

**Authors:** Jie Dong, Jinchao Liu, Xing Li, Qingyou Liang, Xiangying Xu

**Affiliations:** ^1^ Department of Radiation Oncology The Third Affiliated Hospital of Sun Yat‐sen University Guangzhou 510630 P. R. China; ^2^ Analytical and Testing Center South China University of Technology Guangzhou 510640 P. R. China; ^3^ Department of Oncology The Third Affiliated Hospital of Sun Yat‐sen University Guangzhou 510630 P. R. China

**Keywords:** immobilization membranes, poly(ε‐caprolactone), radiotherapy, Young's modulus

## Abstract

Cancer is a leading cause of death in the world. In cancer radiotherapy, immobilization membranes composed of cross‐linked poly(ε‐caprolactone) (PCL) are utilized for patient positioning. A higher‐dimensional stability of the membrane is urgently required to facilitate more accurate radiation dose delivery. It is extremely important to establish the relationship between the degree of crystallinity and the Young's modulus (*E*) because it determines the mechanical properties and can be modulated by crystallinity. When two components of the membrane with different strains are in contact, a gradient region adjacent to the interface is formed and confirmed by attenuated total reflection infrared microscopy. Atomic force microscopy (AFM) and Raman spectroscopy are used to scan the same area in the gradient region (14 µm × 14 µm) to characterize *E* and crystallinity (*X*_Raman_), respectively. This co‐localized method ensures the accuracy of the relationship. Finally, 1764 AFM measurement data are processed and 49 pairs of *E*‐*X*_Raman_ data are obtained. The regression curve shows that *E* monotonically increases with *X*_Raman_. The nonlinearity of the curve may be attributed to the α‐relaxation and cross‐linking of PCL chains. The chemical structure of this material significantly impacts the mechanical properties, thus requiring future investigation.

## Graphical abstract

The nonlinear relationship between the Young's modulus (*E*) and the degree of crystallinity (*X*_Raman_) is established for cross‐linked poly(ε‐caprolactone) (PCL). PCL is used in cancer radiotherapy as an immobilization membrane. Atomic force microscopy and Raman spectroscopy focusing on the same micro‐area are used to obtain *E* and *X*_Raman_, respectively, thus ensuring an accurate relationship. *E* monotonically increases with *X*_Raman_.

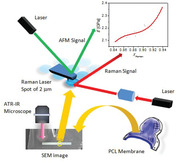

Cancer is now responsible for a leading cause of global death. Based on the global cancer statistics (2018), there were 18.1 million diagnoses and 9.6 million deaths. The incidence and mortality of cancer are rapidly growing worldwide;^[^ ^1^, ^2^ ^]^ thus, cancer continues to pose serious threats to the global population.^[^ ^3^ ^]^

Several treatment modalities are employed to treat cancer, such as surgery, chemotherapy, radiotherapy, and immunotherapy. Radiotherapy, which utilizes ionizing radiation including X‐rays, γ‐rays, and charged particles to kill cancer cells, is used to treat more than half of the cancer patients.^[^ ^4^ ^]^

Immobilization membranes, which are utilized for patient positioning to ensure a precise focus of the ionizing beam on the tumor tissues, are essential medical devices in radiotherapy.^[^ ^5^ ^]^ Cross‐linked poly(ε‐caprolactone) (PCL) is one of the semicrystalline polymers that accounts for 70% of the worldwide polymer production.^[^ ^6^, ^7^ ^]^ PCL is ideal for this membrane because of its mechanical performance, convenience, and biocompatibility.^[^ ^8^ ^]^ The stiffness and damping capacity endows the PCL with excellent mechanical properties.^[^ ^9^, ^10^ ^]^ The stiffness (or rigidity) is determined by the Young's modulus (*E*), which indicates the ability of the material to resist mechanical deformation.^[^ ^11^ ^]^ At present, numerous precision medicine tools have been extensively applied to achieve individualized radiotherapy adaptation and treatment.^[^ ^12^, ^13^, ^14^, ^15^ ^]^ Adaptive radiotherapy is highly encouraged and promoted due to the ability to achieve accurate dose delivery with increased sophistication.^[^ ^14^, ^16^ ^]^ Thus, a higher dimensional stability of the immobilization membrane is urgently required for improved fixation of the patients. Since *E* determines the stiffness, an elaborate manipulation of *E* is extremely important for improving radiotherapy.

The *E* of polymers is significantly influenced and can be modulated by the degree of crystallinity.^[^ ^9^, ^17^, ^18^, ^19^, ^20^, ^21^ ^]^ Therefore, it is essential to establish the relationship between *E* and crystallinity for the membrane. However, this relationship has not yet been elucidated for PCL, particularly for cross‐linked PCL. Although the relationship between the shear modulus (*G*) and crystallinity of linear PCL has been established by simultaneous rheometry and Raman spectrometry,^[^ ^20^, ^22^ ^]^ it is very difficult to convert *G* into *E* in dynamic state. Atomic force microscopy (AFM) enables the simultaneous acquisition of morphological and mechanical data, while Raman spectroscopy functions as a convenient non‐destructive optical method to provide information on the crystallinity.^[^ ^18^, ^20^, ^22^, ^23^, ^24^, ^25^, ^26^, ^27^ ^]^ Herein, both methods, focusing on the same zone of the sample, are employed to establish an accurate relationship between the two parameters. Based on this relationship, *E* can be optimized, which consequently reduces the PCL membrane deformation, thereby facilitating higher precision in adaptive radiotherapy.

The immobilization membrane was produced by extrusion using PCL pellets (CAPA 6500, Perstorp UK Ltd.). The molecular weight (*M*_w_) and polydispersity are ≈8 × 10^4^ and ≈1.8, respectively. PCL was cross‐linked by a Co‐60 source that emitted γ‐ray irradiation at doses of 10–30 kGy. Two small components of the immobilization membrane were drawn in a water bath with 224% and 131% strain. The water bath was set at 70 °C, which is a slightly higher temperature than that of the melting point of PCL (63 °C), to regulate the viscosity of the polymer. Then, the two components were maintained in contact with each other at 25 °C. Therefore, the gradient of crystallinity and *E* was formed in the area adjacent to the interface when the sample was cooling. Attenuated total reflection infrared (ATR‐IR) microscopy was used to rapidly determine the scope of the gradient area due to its ability to characterize the crystallinity of the polymers.^[^ ^28^, ^29^ ^]^ In addition, the aperture of IR microscopes can be programmed to large sizes (tens of micrometers). The relationship between the peak‐area ratio (1470–1458 cm^‐1^) and the crystallinity was established. The bending vibration mode of methylene (CH_2_) shown in **Figure** **1**a strongly correlates with the crystalline phase of PCL. A series of PCL samples with different degrees of crystallinity was prepared by applying different crystallization conditions. The degrees of crystallinity were determined by differential scanning calorimetry (DSC) using a calculation of the enthalpy of fusion.^[^ ^30^ ^]^ Figure 1b shows that the peak‐area ratio of the two peaks monotonically increases with crystallinity. The aperture of the ATR‐IR microscope was set at 50 µm and 16 points (regions) aligned in the direction perpendicular to the interface were manually scanned from the low‐ to the high‐tensile area in ATR mode. Automatic scanning was difficult due to the close contact between the sample and the ATR crystal. All the points are shown in Figure 1d (scanning electron microscopy) image of the entire sample. As shown in Figure 1c, at the 10th point, which is located at the interface, the ratio began to exceed 1:1. This implies that the 10th point (50 µm × 50 µm) is a critical position that may contain composite physical and chemical information, and must thus undergo further AFM and Raman investigations.

**Figure 1:**
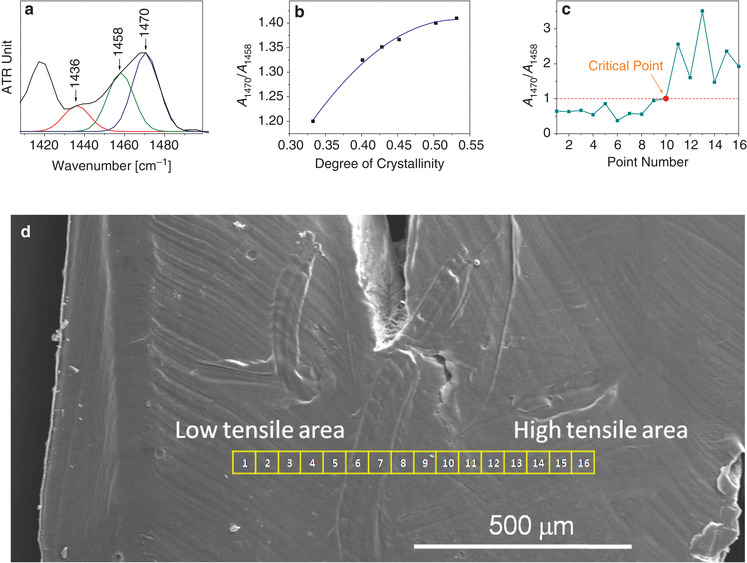
a) Bending vibration mode of methylene (CH_2_) for cross‐linked PCL in the ATR‐IR spectrum. b) Relationship between the peak‐area ratios of peaks 1470–1458 cm^‐1^ in the ATR‐IR spectrum and the degree of crystallinity of cross‐linked PCL. c) Relationship between the peak‐area ratio of peaks 1470–1458 cm^‐1^ and scanning point number on the sample using ATR‐IR microscopy, with the 10th point being a critical point at which the ratio begins to exceed 1:1. d) SEM image of the entire sample with ATR‐IR microscopy scanning points numbered 1–16, depicted by yellow boxes (50 µm × 50 µm for each box, representative of ATR‐IR microscope aperture). The scanning direction is perpendicular to the interface formed between the low‐ and high‐tensile areas. The 10th point is exactly at the interface.

Then, AFM mapping of the topography and *E* of the 10th point were performed. The mapping area in the center of this point was fixed at 40 µm × 40 µm and the mapping step was 0.32 µm. The topography in **Figure** **2**a shows that there is a relatively flat region in the center of the mapping area that is suitable for Raman analysis. *E* was determined in PeakForce QNM mode with the oscillation frequency of the cantilever in the *z*‐direction being 2.0 kHz. Figure 2b indicates that *E* decreases from the high‐ to the low‐tensile area due to different crystallization behavior caused by the two stresses used in the sample preparation.^[^ ^7^ ^]^

**Figure 2:**
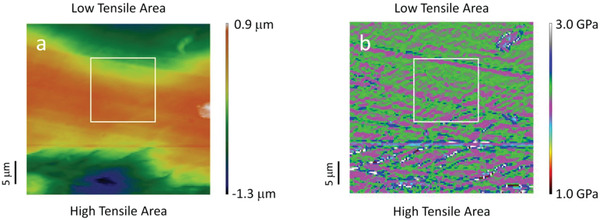
AFM mapping results with the dimension 40 µm × 40 µm in the 10th point of ATR‐IR scanning. The white box is representative of the Raman mapping area (14 µm × 14 µm). a) AFM mapping of topography with a relatively flat area in the center. b) AFM mapping of the Young's modulus with the modulus gradient from the high‐ to the low‐tensile area.

After AFM imaging, the degree of crystallinity was determined using Raman spectroscopy. The Raman spectrum originates from Raman scattering, an inelastic scattering light that reflects the change of polarity of the material molecules.^[^ ^31^ ^]^ The first step required to calculate the crystallinity was to assign the Raman peaks of PCL.^[^ ^26^ ^]^ The weak Raman peak at 1720–1740 cm^‐1^ represents the carbonyl (C=O) stretching vibration. The peaks at ≈1444 cm^‐1^ are assigned to the bending vibration of methylene (CH_2_), while the peaks at 1305 and 1284 cm^‐1^ are assigned to the CH_2_ twisting vibration. The skeletal stretching vibrations encompass the peaks lower than 1200 cm^‐1^, where 1109, 1037, and 1064 cm^‐1^ are assigned to the C—C symmetric stretching mode, C—C asymmetric stretching mode, and C—O stretching mode, respectively.^[^ ^26^, ^32^ ^]^ All of the above‐mentioned peaks are related to the crystallinity.^[^ ^17^, ^25^ ^]^

In the three‐phase model of semicrystalline polymers, the interphase also plays an important role in the mechanical properties.^[^ ^33^, ^34^, ^35^, ^36^ ^]^ The crystallinity determined by Raman spectroscopy (*X*_Raman_) is attributed to both the crystal phase (*X*_c_) and the interphase (*X*_i_).^[^ ^9^ ^]^ Consequently, it is rational that *X*_Raman_ is proportional to the crystallinity determined by the density method (*X*_Density_) but not to that determined by the DSC method (*X*_DSC_), which only reflects the contribution of the crystal phase.^[^ ^9^ ^]^ A classical method proposed by Prof. Strobl provided a detailed description of the calculation of crystallinity in polyethylene (PE) using Raman spectroscopy.^[^ ^25^ ^]^ This method can also be employed for PCL, because the molecular and unit cell structures of PCL are both similar to those of PE.^[^ ^27^, ^37^, ^38^ ^]^ In brief, the peaks between 1250 and 1350 cm^‐1^ are fitted in the Lorentz profile shown in **Figure** **3**a. The peaks at 1283 and 1305 cm^‐1^ correlate with the crystalline and intermediate phases, as evidenced by their characteristic sharp shape. The peak at 1283 cm^‐1^ can be further attributed to the single‐chain mode.^[^ ^26^ ^]^ Since the peak at 1310 cm^‐1^ is broad, it can be used as an indicator of the amorphous phase.^[^ ^25^ ^]^ The relationship between *X*_a_ and the sum of the peak areas at 1250–1350 cm^‐1^ (*A*_1250–1350_) and 1310 cm^‐1^ (*A*_1310_) can be expressed by Equation (1)

(1) $$X_{\text{a}}=\;\;\frac{A_{1310}}{A_{1250-1350}}$$

**Figure 3:**
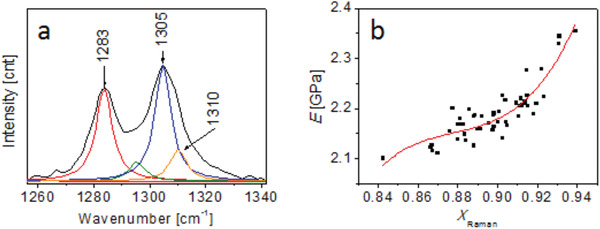
a) Fitted peaks between 1250 and 1350 cm^‐1^ in the Raman spectrum. b) The regression curve of the Young's modulus (*E*) versus the degree of crystallinity (*X*_Raman_) for the immobilization membrane.

Therefore, *X*_Raman_ can easily be obtained by Equation (2)

(2) $$X_{\text{Raman}}=\;\;X_{\text{c}}+\;\;X_{\text{i}}=\;\;1\;\;-\;\;X_{\text{a}}$$

In the Raman analysis, a 785 nm laser, with a laser spot diameter of ≈2 µm and a ×50 objective lens, was used. A 14 µm × 14 µm region in the center of AFM image was selected for Raman analysis because it was relatively flat. Thus, 49 (7 × 7) data points of Raman spectra were generated. Given that the step used was 0.32 µm, the sum of the area of every 36 (6 × 6) square units in AFM is approximately equivalent to the area of the laser spot of Raman. The average value of these 36 measurements for the Young's modulus by AFM corresponds to one measurement of crystallinity by Raman. Finally, 1764 (36 × 49) AFM measurement data were processed. Since 49 pairs of the Young's modulus and crystallinity data were prepared, the fitting curve shown in Figure 3b was plotted with the regression equation given in Equation (3)

(3) $$E\;\;=\;\;709X^{3}-\;\;1869X^{2}+\;\;1642X\;\;-\;\;479\;\left(R^{2}=\;\;0.7859\right)$$

where, *E* (GPa) and *X* refer to the Young's modulus and *X*_Raman_, respectively.

The curve reveals that the Young's modulus monotonically increases with the degree of crystallinity between the range from 0.84 to 0.94. However, despite the prior anticipation of linearity, the correlation is not linear. One possible reason for the deviation from linearity is the relaxation of polymer chains.^[^ ^9^ ^]^ The temperatures of the α and α′ relaxation of PCL are 220 and 270 K, respectively,^[^ ^39^ ^]^ which are lower than the room temperature (298 K). The α‐relaxation, which also increases with crystallinity,^[^ ^40^ ^]^ involves rigidity loss in the crystalline phase. Thus, the relaxation has a comprehensive effect on the regression curve. Another reason is speculated to be cross‐linking. The network formed by cross‐linked chains can modify the mechanical coupling representing the stress concentration of hard and soft phases, which is inversely related to the modulus.^[^ ^9^ ^]^ The disorder in the interlamellar regions can be increased by cross‐linking and may thus impact such coupling.^[^ ^41^ ^]^

In conclusion, the cross‐linked PCL is applied as an immobilization membrane in adaptive radiotherapy. The relationship between Young's modulus and crystallinity is essential for improving therapeutic precision. Hence, this relationship was established using AFM and Raman spectroscopy focusing on the same micro‐area, thereby ensuring the accuracy of the relationship. The Young's modulus monotonically increased as the degree of crystallinity increased from 0.84 to 0.94. The regression equation was not linear, which is potentially attributed to the fact that the α‐relaxation interacts with the crystallinity and the polymer network impacts the mechanical coupling. The findings of this study provide a foundation for further investigation of the influence of the Young's modulus on the outcome of adaptive radiotherapy and the optimization of the modulus. Moreover, exploring the effect of the chemical structures of cross‐linked polymers on the mechanical properties also plays a vital role in achieving enhanced precision, which is required for reducing the mortality of cancer patients.^[^ ^42^ ^]^

## Conflict of Interest

The authors declare no conflict of interest.
